# Documenting the mental health climate in correctional work and the realities of suicide

**DOI:** 10.3389/fpsyg.2022.1026821

**Published:** 2023-01-04

**Authors:** Matthew S. Johnston, Rosemary Ricciardelli

**Affiliations:** School of Maritime Studies, Fisheries and Marine Institute, Memorial University of Newfoundland, St. John’s, NL, Canada

**Keywords:** correctional workers, suicide, stigma, occupational culture, intervention

## Abstract

Public safety personnel are at an elevated risk for suicidal thoughts and behaviors relative to the general public. Correctional workers in particular report some of the highest prevalence of suicidal thoughts and behaviors. To better understand this phenomenon, the current study draws on qualitative, open-ended survey response data (*n* = 94) that explores three distinct themes (occupational environment, lack of support, social silence) and how entrenched notions of mental health stigma and occupational culture inform how Canadian correctional workers understand their experiences with suicidal thoughts and behaviors. We conclude with a brief discussion of the research and policy implications, with an emphasis on mobilizing efforts to normalize mental health discussion in correctional workplaces, bolstering peer support resources, and collaboration, and assessing the limited organizational supports available to struggling staff.

## Introduction

Given that more than 700,000 people die by suicide every year, suicide is a global health problem (World Health Organization [WHO], 2021). Public safety personnel, such as police officers, correctional workers, paramedics, and firefighters, are at an elevated risk for suicidal thoughts and behaviors relative to the general public (Stack and Tsoudis, 1997; Stanley et al., 2016; Milner et al., 2017; Violanti, 2017; Carleton et al., 2018b; Grassi et al., 2019; Vigil et al., 2019). This predisposition is due, in part, to their stressful and potentially psychologically traumatic work environments, shift work and long working hours, and pervasive unpredictability of threat and severe violence (Carleton et al., 2021). One study with a large and diverse sample found that, in Canada, correctional workers reported past-year or lifetime suicidal ideation (7.0%, 26.6%), planning (2.6%, 11.9%), and attempts (n/a, 5.2%), with a prevalence being considerably higher than general population estimates (Carleton et al., 2021). Within a similar dataset in an earlier study, 54.6% of correctional workers screened positive for one or more mental health disorders (Carleton et al., 2018a).

Other challenges associated with mental health disorder symptoms and/or difficult social and relational factors (i.e., interpersonal conflict, child abuse, low socio-economic status, bereavement, bullying), alongside frequent exposure to potentially psychologically traumatic events such as violence, harassment, abuses, suicide, threats, or other unhealthy workplace conditions (i.e., overcrowding, infectious disease), may aggravate correctional workers’ tendencies toward suicide behavior and thoughts (Turner et al., 2018; Carleton et al., 2019; Ricciardelli et al., 2020; Genest et al., 2021; Lerman et al., 2022). The intersection of personal and social factors in understandings of suicide behavior such as stressful work conditions (Violanti et al., 2008; Genest et al., 2021), prevalence of mental health disorders (Violanti et al., 2008), including substance use disorders (Henderson et al., 2016), and family conflict/relationship concerns (Triplett et al., 1999; Dixon, 2021; Genest et al., 2021; Higgins et al., 2022) can coincide with difficult occupational social climates. More specifically, Genest et al. (2021) found that correctional workers’ experiences with marital or family problems and difficulties at work (i.e., bullying) sometimes intensified or precipitated suicide thoughts and behaviors, while having children and a partner tended to act as protective factors for those presenting with ideation.

Correctional workers must possess and apply significant knowledge around suicide prevention and risk factors to protect and help criminalized persons and many correctional workers are receptive to increase their knowledge of critical mental health issues (Penn et al., 2005), yet paradoxically, mental health stigma remains and permeates their workplace culture (Johnston et al., 2021, 2022b). The general stigma surrounding mental health disorders and suicide behaviors in correctional work may impact coping mechanisms and responses (Velazquez and Hernandez, 2019; Ricciardelli et al., 2020), thus warrants considerably more scholarly and empirical attention. Genest et al. (2021) found that some participants experiencing suicide thoughts and behaviors would seek help from professionals, such as their family doctors, a psychologist, or Employee Assistance Program (EAP), but the lack of perceived organizational supports and recognition of the issues by the employers also hindered the treatment and help-seeking process.

In their online survey study of 440 participants, Trounson et al. (2016) found that correctional officer (*n* = 37) perceptions of work-related environmental adversity were significantly higher than the perceptions of people employed across of wide range of other general occupational roles. To this point, Frost and Monteiro’s (2020) case study found that the interaction of occupational and personal risk factors, tied to occupational work culture, best explained suicide among 20 correctional officers. Indeed, challenges can present in a profession where staff must constantly manage their difficult emotions and circumstances in context-sensitive, professional, appropriate ways, as well as the emotions and circumstances of others, in order to maintain the integrity of their institution (Liebling et al., 2011).

This research note is an extension of a larger, theoretical study (Johnston et al., 2022a) documenting Canadian correctional workers’ narrations of mental distress and suicide behaviors, thoughts, and experiences. There, we explored how correctional workers negotiated and survived these ordeals through varying agencies that were sometimes described as intentioned and directed, while at other times more susceptible to the influence (and intervention) of external forces (i.e., spiritual, systemic, another individual). In the current study, we answer the following research question: How do correctional workers’ experiences of stigma and mental health resistant occupational cultures inform how they understand their suicide thoughts and behaviors? We conclude with a brief discussion of the policy directions, changes in occupational culture, and resources that correctional services can implement to better support the mental health treatment, awareness, and well-being of correctional workers.

## Methods

Using a web-based self-report survey available in English or French, we surveyed correctional employees working in Canadian provincial or territorial correctional services about their work life, occupational roles and responsibilities, peer networks, mental health concerns, and other experiences. Ministerial correctional and union representatives contacted participants *via* their email listserv to invite them to participate in an anonymous and confidential online survey in 2018 and 2019. All surveys closed prior to/or at the onset of the COVID-19 pandemic. After receiving the circulated survey email, interested respondents were provided with a link that directed them to the project consent form, research information page, and survey. Participation was voluntary and the survey could be completed during paid work hours. If desired, participants could choose to finish the survey over multiple sessions. Research ethics boards at the University of Regina and Memorial University of Newfoundland approved the study.

In this research note, we analyze qualitative, open-ended survey responses from 94 participants^1^ who responded to the item, “Please include any additional comments you may have about the section on suicide” from the provinces of Manitoba (*n* = 39), Saskatchewan (*n* = 36), Nova Scotia (*n* = 9), New Brunswick (*n* = 3), Newfoundland (*n* = 3), and the Yukon Territory (*n* = 4). Respondent quotations that were included in our other study (Johnston et al., 2022a) have been excluded from being quoted in the results section of the current study (*n* = 24), though all 94 responses were considered when structuring and capturing the broader themes of the analysis. Participants answered the open-ended question after responding to six close-ended items requesting information about lifetime and past year suicidal ideation, attempts, and planning.

Respondents are comprised of many different types of correctional workers, including correctional and probation officers, managers, administrators, nurses, teachers, and other employees within community, administrative, and institutional correctional services. Given that the sample is small, and the topic is highly stigmatized and sensitive, we present few demographics of the respondents in Table 1, ensuring that we protect respondent privacy.

**TABLE 1:** Select participant demographics.

Variable	*n* (%)
**Occupation**	
Correctional officer	60 (64%)
Institutional other	17 (18%)
Probation officer	12 (13%)
Community/Non-institutional other	5 (5%)
**Gender**	
Man	54 (57%)
Woman	37 (39%)
No response or other	3 (3%)
**Education level**	
No post secondary degree	30 (32%)
Completed 2–3 year degree	22 (23%)
Completed 4 year degree or greater	36 (38%)
Other	6 (6%)
**Marital status**	
Partnered	58 (62%)
Not partnered	30 (32%)
Other/No response	6 (6%)

This study was guided by a realist lens of inquiry. There are many different approaches to realism, but this ontological and epistemological orientation generally holds that metaphysical reality exists independent of individual perception, and that truth, or the idea that we can know the world as it exists in and of itself, is possible (Bonino et al., 2014). Employing NVivo, the second author (principal investigator) and other research assistants initially coded the data using a constructed semi-grounded emergent theme approach that involved transforming codes into primary, secondary, and tertiary themes (Glaser and Strauss, 1967; Charmaz, 2014). We did not know what themes would emerge from the data and we framed the study according to what the data revealed theoretically but did not create any theory (e.g., we reflected on our theoretical knowledge to conceptualize the study); thus we call our approach semi-grounded (see Ricciardelli et al., 2010). Grounded theory is generally helpful to reset dominant ideas in the literature because it is inductive and guided largely by data. Correctional workers in Canada are often depicted critically in more ideologically driven criminological literature (e.g., Walby et al., 2018), and since the study of correctional workers through the lens of correctional workers is an emerging field of study in Canada, we believe this approach to data coding and analysis added value to this research.

The first author then read through, reviewed the coding, and categorized predominant themes found across all 94 responses. The reviewing of the coding—the use of axial and open coding—was necessary and beneficial, allowing us to be fully immersed in all of the data, and for more nuances to emerge from the data. We coded all data – not just selective coding – to gain a full picture of the phenomenon of suicide among correctional workers. The data were grouped into one document and both authors then collaboratively discussed the themes to get a sense of the data as a whole and to identify significant themes across responses (Corbin and Strauss, 2015), as well as decide how to best represent the key themes. It is important to note that while we coded all 94 responses to produce *overarching* themes, these data captured in this study focuses on the words of 41 participants who fell under the subthemes of occupational environment, social support and silence within the greater theme of suicide. This research note unpacks these subthemes from a larger dataset. It is a nuanced analysis that complements that found in Johnston et al. (2022a). We believe nuanced unpacking of themes around suicide is essential given the very real phenomenon of suicide – a permanent choice too often – striking correctional work in Canada and globally (Genest et al., 2021). Our results section showcases selected and representative quotations that illustrate the concrete themes and voices of respondents. Without compromising meaning or tone or impacting vernacular, the quotations we cite have undergone minor edits to correct spelling and grammar, and enhance readability.

## Results

The following analysis is divided into three themes: occupational environment, lack of support, and social silence. More specifically, we explore how these distinct themes inform participant understandings of their experiences with suicide thoughts and behaviors.

### Occupational environment

In total, 29 respondents identified that their occupational environment was tied to their understandings of their suicide thoughts and behaviors. The pressures and demands of correctional work are strenuous and can increase in intensity at any point, depending on the fluctuating needs of the worker’s institution. Participant 491, from Manitoba, described quite vividly how the accumulation of responsibility and numerous duties can lead to overwhelming experiences with fatigue and stress:

Dealing with the daily pressure of being so short staffed at work due to cutbacks and having to support an extremely large staff, handling 100–120 emails a day, fielding multiple in person staff requests for assistance, being given multiple additional admin tasks, working at adrenalin pace trying to keep up with the excessive workload, plus training and supervising temporary/revolving employee placements (four in the last year) and unsuccessfully attempting to find my own coverage for vacation time I want to take, in addition the unsafe environment inside and outside work as described previously has been overwhelming. It has resulted in health issues, insomnia, violent nightmares that wake me up at night, and yes…the battle with the s-word.

Participant 491’s words draw our attention to how human resource shortages in some correctional environments have caused staff to take on extra and “excessive” responsibilities and duties to ensure that their institution runs smoothly. Left with no workers to cover their request for time off, participant 491 laments over not being able to take a much-needed break from the intensified routine and “unsafe” working environment. In consequence, they began experiencing health problems, disturbances, as well as suicide-related feelings they had to “battle” in the midst of being utterly overwhelmed at work.

Reflecting on how social context influences pathways to suicide more explicitly, 12 respondents commented further on the role of occupational environments. On this topic, two inter-related central themes emerged; perceived lack of support and social silence, which we now discuss.

### Lack of support

Nine (9) respondents discussed how perceived lack of support (e.g., an absence of effective mental health interventions in response to occupational stress injuries) influenced pathways to suicide. In this sense, suicide thoughts and behaviors are shaped by the perceived social climate – an effect of lack of availability (or engagement with) support interventions prior to negative feelings or experiences cumulating into suicide thoughts and/or behaviors: “Nothing is done by the employer to assist staff and yet it is a known issue with correctional officers and front line staff. I was off work for 1.5 years and received zero assistance from work or the union with regards to PTSD and depression. Work actually fought my workers compensation PTSD claim” (participant 404, from Manitoba). This participant describes their frustration with having to fight on their own to acquire compensation for their mental health injury.

Some participants noted that support systems were in place (e.g., employee assistance programs), but failed to provide the level of support needed to effectively address these stress injuries: “The EFAP can go fuck themselves. They make it harder to access care by sending you to blow out priced services that are not adequate” (participant 365, from Saskatchewan). Others expressed feeling as though the organization was apathetic or even hostile to mental health and suicidal concerns among staff, noting they received minimal assistance and/or concerns were ignored: “Senior management was aware of my suicidal actions and ignored to do anything” (participant 478, from Manitoba).

Lack of support was also expressed in terms of negative reactions toward workers using the resources in place to cope with mental health and suicide-related concerns, such as leave of absence from work. One respondent, participant 34 from Saskatchewan, described how, following a suicide attempt, they took a period of leave, but were met with negative and hostile reactions upon their return: “Upon returning to work I was then harassed and intimidated by a manager who was unaware of the reasons I was on sick leave…These two managers did not ask if I was okay or feeling better from my illness but instead attacked me on my absence.” Though the exact reason for their use of leave was not known at work, they felt they were treated harshly, with suspicion, and devoid of empathy or compassion, which compounded their already “distress[ing]” experiences and had lasting impacts on their “ability to function and overall performance in my work duties.” This finding suggests that negative connotations associated with use of leave within the work environment (e.g., due to the problem of staff shortages) may result in negative social repercussions when staff use leave for personal health reasons.

### Social silence

Another related way the social environment affected experiences toward and of suicide was the silence and stigma surrounding the topic. Specifically, three (3) respondents expressed that they are not inclined to speak openly about the topic of suicide given the “stigma attached to the subject” (participant 12, from Nova Scotia). As participant 256 from Manitoba stated, “It’s not something you talk about.” Hesitancy to discuss seemingly personal matters such as suicide may be partially shaped by masculinized norms in occupational culture (e.g., demonstrating strength and machismo; Crawley and Crawley, 2008), as well as sentiments of mistrust that often characterize correctional environments (Arnold et al., 2007). As participant 127 from Manitoba expressed, “I don’t trust anyone to keep this quiet, if I ask for help it will get back to my supervisor or co-workers.”

Across these responses was a sense that the socio-occupational environment was experienced negatively around concerns involving suicide, cumulating in perceived lack of support, stigma, and social silence. The silencing effect may lead correctional workers to internalize their struggles, become isolated, and thus rely on, and search for, other forms of—sometimes unpredictable—support mechanisms to survive, cope, and manage.

## Discussion and conclusion

This study set out to explore three distinct themes germane to the realities of suicide thoughts and behaviors in correctional work (occupational environment, lack of support, social silence). More specifically, data revealed how entrenched notions of mental health stigma and occupational culture inform how Canadian correctional workers understand their experiences with suicide thoughts and behaviors. Before discussing these findings in more detail, we first couch them in the context of the study’s limitations.

We note four methodological limitations of the current study. First, we can only analyze the information presented to us anonymously in the survey by participants; thus, we could not probe respondents for additional information or clarity. Second, the sampling frame, due to the overlap between institutional and union listservs, cannot be determined. Therefore, we cannot confirm a response rate for survey participants. In this vein, we make no claims to generalizability, especially given the smaller sample sizes in some provinces and territories. Third, this study focused on experiences of suicide thoughts and behaviors, which was operationalized as action or thoughts toward death by suicide, and contrary to notions of agency, control, and well-being (Johnston et al., 2022a). Thus, this research excludes related but potentially departing experiences of self-injury, which may encompass experiences of self-harm that do not move toward the end of life via suicide, and in some cases, embody more agency (Claréus et al., 2021). Future research should investigate the phenomena of self-injurious practices among correctional workers in Canada and internationally, as this work could distinguish more clearly between thoughts or actions that move toward end-of-life and actions that may reflect other various and nuanced degrees of self-harm. Fourth, though the theme was less relevant to our research question and not as prevalent, five (5) respondents identified explicitly how their suicide thoughts and behaviors were tied to factors outside of work (i.e., death of loved one, health concern). Future research should also consider in-depth how life stressors outside of work shape correctional workers’ pathways to suicide thoughts, behaviors, or self-injury.

That said, in the context of correctional work, occupational environment, social silence, and perceived lack of support were found to shape pathways toward suicide behaviors and thoughts. Our participants expressed how the accumulation of occupational responsibilities can be overwhelming, with many having struggled with ideation. Moreover, as a result of occupational and social pressures, staff who are suffering mental distress may feel they are unable to speak up or access help out of fear of adverse social repercussions, which aligns with broader studies on the mental health cultures and paradigms of correctional work in Canada (Johnston et al., 2021, 2022b).

Thus, the culture remains inflexible and avoidant of discussion of mental health. This finding is also consistent with prior research that highlights how public safety occupational environments and mental health stigma can impact staff coping mechanisms and responses (Velazquez and Hernandez, 2019; Ricciardelli et al., 2020). The challenge, however, is that without intervention or support, mental distress may cumulate into thoughts toward the end of their life—and too often such thoughts are acted upon. Moreover, intervention and support are difficult to provide given the vast stigma (Johnston et al., 2022b) – in society, it remains offensive to suggest someone requires mental health support in many contexts. As such, the stigma prevails and hinders treatment and help-seeking. This study therefore contributes to the broader literature on correctional workers and mental health by showcasing how the permeation of stigma in occupational cultures can suppress open discussion about mental health and available resources to such an extent that even suicide thoughts and behaviors become internally normalized or self-regulated, which we argue is dangerous and not conducive to a healthy workforce.

Participants generally felt their employer failed to support their mental health. Here, a challenge arises, as the employer appears in tension. Providing supports may be interpreted as a “band aid” solution and employees avoid using them due to scepticism and distrust, or such supports can be perceived as responding to employee needs, but due to hesitancy and concerns around stigma, may still be avoided. Thus, the employer remains in a difficult position where action intended to support staff may be misconstrued given that the correctional culture is laced with stigma around suicide and mental health treatment seeking, as well as in public safety professions more broadly (see Ricciardelli et al., 2020).

Thus, ways forward need to be designed and implemented that recognize the limitations of supports imposed by occupational culture and the vulnerabilities felt and lived by—especially frontline—employees. Suicide can affect nearly every person who enters a prison, but correctional workers, through peer-support programs, can play a critical role in suicide prevention (Diagle et al., 2007). Diagle et al. (2007) note how correctional systems differ in how they include (or not) other partners of suicide prevention, such as correctional officers, other employees, spiritual care workers, and community workers. Lerman et al. (2022) recently found that institutional factors, such as the quality of perceived management and supervision, can play a protective role and moderate the deleterious effects of prison work and mental health strain and pain. Hence, more intervention, peer support collaboration and resources, preventative discussion, and simply overall acknowledgment of mental health is required to start making small strides to change the culture, centralize mental health, and encourage a healthier workforce to emerge. However, to do this with success, more research into employee culture in correctional spaces is necessary. A deeper, in-depth understanding of the culture among staff will illuminate avenues to change how mental health is interpreted within that culture. Nevertheless, change requires time, is incremental, and our hope is that recognizing the context around suicide in correctional work can lead to fruitful discussions on how to make change possible.

## Data availability statement

The datasets presented in this article are not readily available due to confidentiality and privacy. Requests to access the datasets should be directed to RR, rricciardell@mun.ca.

## Ethics statement

The studies involving human participants were reviewed and approved by the Memorial University of Newfoundland and University of Regina Research Ethics Boards. The patients/participants provided their written informed consent to participate in this study.

## Author contributions

MJ drafted the manuscript. RR collected the data, supervised the writing of the manuscript, and revised the manuscript. Both authors contributed to the article and approved the submitted version.

## Footnotes

1 A total of 1999 correctional workers completed the survey, and of these people, 94 responded to the question under study.
